# Myc-Dependent Genome Instability and Lifespan in *Drosophila*

**DOI:** 10.1371/journal.pone.0074641

**Published:** 2013-09-06

**Authors:** Christina Greer, Moonsook Lee, Maaike Westerhof, Brandon Milholland, Rebecca Spokony, Jan Vijg, Julie Secombe

**Affiliations:** 1 Department of Genetics, Albert Einstein College of Medicine, Bronx, New York, United States of America; 2 Department of Human Genetics, The University of Chicago, Knapp Center for Biomedical Discovery, Chicago, Illinois, United States of America; Lancaster University, United Kingdom

## Abstract

The Myc family of transcription factors are key regulators of cell growth and proliferation that are dysregulated in a large number of human cancers. When overexpressed, Myc family proteins also cause genomic instability, a hallmark of both transformed and aging cells. Using an *in vivo lacZ* mutation reporter, we show that overexpression of Myc in *Drosophila* increases the frequency of large genome rearrangements associated with erroneous repair of DNA double-strand breaks (DSBs). In addition, we find that overexpression of Myc shortens adult lifespan and, conversely, that Myc haploinsufficiency reduces mutation load and extends lifespan. Our data provide the first evidence that Myc may act as a pro-aging factor, possibly through its ability to greatly increase genome instability.

## Introduction

The Myc family of oncoproteins (c-Myc, N-Myc and L-Myc in vertebrates) are basic helix-loop-helix zipper (bHLHZ) transcription factors that are highly conserved regulators of growth and development [1], [2]. The canonical means by which Myc proteins regulate gene expression is via their ability to form heterodimers with the small, ubiquitously expressed, bHLHZ protein Max [1], [3]. Myc-Max heterodimers bind to E-box sequences (CACGTG and related DNA sequences) and recruit additional co-activator proteins to regulate the transcription of genes involved in a number of cellular processes, including cell growth and proliferation [4]. More recently, Myc has also been shown to function independently of Max, although these activities remain far less characterized. For example, c-Myc can directly promote DNA replication in mammalian cells and induce cell competition and apoptosis in *Drosophila* in the absence of Max [5], [6]. Myc family proteins can therefore affect a number of cellular processes and can act via more than one molecular mechanism.

Key to its capacity to transform cells when overexpressed is Myc’s ability to induce cell growth and proliferation [7]. In addition, Myc overexpression in mammalian cells increases the frequency of DNA double-strand breaks (DSBs) that are associated with genomic instability [8]–[15]. Because genomic instability is a hallmark of cancer [16], the fact that Myc family proteins can promote this in addition to growth and proliferation may underlie the observation that patients with higher levels of Myc have a poorer prognosis than those with lower levels of Myc [15], [17].

Mutations caused by the misrepair of DSBs are a particularly deleterious class of mutation and are associated with neoplastic transformation and may also contribute to aging [18]–[22]. Consistent with the observed accumulation of DSB-induced lesions and aging, mutations in genes encoding DSB repair proteins can cause phenotypes that are often associated with premature aging in flies, mice and humans [23]. While c-Myc overexpression can cause DSBs and increase genomic instability in mammalian cells [6], [24]–[26], a possible link with aging and lifespan has not been examined. Moreover, the important question of what role (if any) endogenous Myc plays in influencing cellular mutation load and lifespan has not been addressed.

Here we generate a genetically amenable *Drosophila* model to quantitatively examine the effects of Myc levels on genome instability and on lifespan. *Drosophila* encodes a single, essential Myc ortholog, Myc, which is highly functionally conserved with its mammalian paralogs [2]. Using *Drosophila* strains that harbor an *in vivo lacZ* mutation reporter transgene [27]–[29], we show that overexpression of *Drosophila* Myc increases the number of DSBs, doubles somatic mutation load, and reduces lifespan. Conversely, *myc* haploinsufficiency reduces spontaneous mutation load and increases lifespan. This provides the first evidence that endogenous Myc may play a crucial role in modulating lifespan, possibly by influencing levels of genome instability.

## Materials and Methods

### *Drosophila* Stocks

All fly stocks were maintained at 22–25°C on standard medium unless otherwise indicated. *myc^4^*, UAS-Myc, hs-FLP; Act>CD2>Gal4, UAS-GFP and the *lacZ* reporter transgenes have been previously described [29]–[31]. The Act^TS^ stock was generated by combining Tubulin-Gal80^TS^ (2^nd^ chromosome; Bloomington stock center) with Actin-Gal4 (3^rd^ chromosome; Bloomington stock center). Apterous-Gal4 (ap-Gal4) was obtained from the Bloomington stock center. The UAS-p35 strain was obtained from Dr. Bruce Edgar (University of Heidelberg) and is a *P* element insertion on the 3rd chromosome that has been used previously to inhibit cell death (e.g. Jiang et al. [32]).

To construct *myc:GFP* flies, P[acman] BAC CH321-88A16, covering 72 kb of genomic DNA from the X chromosome (3224568 to 3296675) [33] was used to drive Myc expression. Recombineering was performed to tag Myc in frame with the coding region as described in Jungreis et al., [34] using plasmids containing the recombineering plasmids (Donald Court, National Cancer Institute, Frederick, MD) [35]. The stop codon for *myc* was replaced with the coding sequence for superfolder EGFP codon-optimized for *Drosophila* [36] followed by sequence coding for a flag tag, a Precision site, a TEV site and the biotin ligase recognition peptide. The tagged construct was injected into a stock containing an attP site on the 3^rd^ chromosome (Bloomington Stock Number 24871). Integration was marked with *w* ^+^. Transformants were validated by PCR amplifying across the attL and attR integration events as described previously by Venken et al. [37], and the final exon-tag junction (myc specific primer: ATACGATCGAGAAGCGCAAT, EGFP reverse primer: CGATGTTTCGCTTGGTGG-TCGAAT). PCR products of the expected sizes were found to support insertion of the BAC at the attP site and the retention of the tag. The line is available from the Bloomington Stock Center, stock number 38633.

### LacZ Mutation Assays

To determine *lacZ* mutation frequency in larvae, hs-FLP; UAS-Myc (or hs-FLP control) females were crossed to *lacZ* #2 (or *lacZ* #9); Actin>CD2>Gal4, UAS-GFP males. 1^st^ instar larvae (24–48 hrs AED) were heat shocked at 37°C for 45 minutes to induce recombination in ∼90% of cells and 3^rd^ instar larvae were collected 3 days later. Larvae were sexed and then stored at −80°C until assays were carried out as described previously [27], [29]. 30 larvae were used for each *lacZ* experiment. For *lacZ* mutation frequency in adults, Tub-Gal80^TS^; Actin-Gal4/TM6B females were crossed to *lacZ* #2; UAS-Myc (or *lacZ* #2; +) males at 18°C. From this cross, Tub-Gal80^TS^/*lacZ* #2; Actin-Gal4/UAS-Myc (and Tub-Gal80^TS^/*lacZ* #2; Actin-Gal4/+) females were placed at 25°C or 29°C to induce expression of Myc for 10 or 5 days, respectively. Adults were stored at −80°C until *lacZ* assays were carried out. 50 adult flies were used per *lacZ* experiment and 3–5 experiments were carried out for each genotype. The mutation frequency is the ratio of colonies growing on the selective plate vs. the total number of recovered plasmids from the DNA sample (as measured on the titer plate). Hence, mutation frequencies as determined with this system reflect a ratio and do not depend on the amount of DNA. They are expressed on a per locus basis as the number of mutant *lacZ* copies for a given number of *lacZ* copies isolated from the *in vivo* situation.

Categorizing *lacZ* mutations into “point mutations” and “genome rearrangements” was carried out as previously described [27], [29]. Candidate genome rearrangements were sequenced using *lacZ* primers as previously described [27] and breakpoints were mapped to the *Drosophila* genome sequence. Briefly, the Sanger sequences for each sample were aligned against the sequence of the *LacZ* construct using blastn from the NCBI Blast+ toolkit [38], version 2.2.26. The top hit for each sequence was taken, and BEDTools [39] was used to combine the alignments; the resulting intervals of the construct present in the Sanger sequences were reported. In addition, alignments whose start sites were at a position after their end sites were reported as inversions. Finally, the Sanger sequences were aligned to the organism genome with blastn and the top hit, if any was found, for each sequence was reported, along with its position in the construct as inferred from partial alignment of the Sanger sequence with the construct.

### Larval Antibody Staining and Western Analyses

For antibody staining, third instar larvae were dissected, fixed and stained as previously described [40]. Anti-γ-H2A.X was obtained from Active Motif and used at 1∶500. Secondary antibodies were obtained from Invitrogen. For Western bot analyses, larval wing imaginal discs or adult heads of the appropriate genotype were homogenized in SDS load buffer, sonicated and run on a 4–12% gel (Invitrogen). Western blots were carried out using standard procedures and Myc protein detected using anti-Myc mouse monoclonal antibody that has been described previously [41] (used at 1∶500). Anti-gamma-tubulin antibody was obtained from Sigma and used at 1∶2000. Westerns were analyzed using infrared conjugated secondary antibodies (LiCOR) and Odyssey scanner and software (v3.0) to quantitate levels of protein using γ-tubulin to normalize loading. Quantitation was carried out from three independent Westerns per genotype using samples from independent crosses.

### Lifespan Assays

Flies of the specified genotype were placed at low density (20–25 flies per vial) at the temperature indicated (with 50% humidity and 12 hour light/dark cycle). The number of dead flies was scored every 2–3 days and the living flies transferred to new food. Lifespan experiments were carried out at least three times from independent parents using ∼100 flies per genotype. Representative lifespan experiments are shown in the Figures. The *myc^4^* null allele and the myc:GFP transgene were backcrossed into the *w^1118^* background for five generations to limit genetic background effects. The UAS-Myc and *lacZ* transgenes were both generated in *w^1118^*.

### Statistical Analyses

The statistical significance of *lacZ* mutation frequency data and γ-H2A.X staining were determined using a student’s t-test (Microsoft excel). Statistical significance of lifespan data were determined with Log-rank (Mantel-cox) test using Prism (GraphPad software).

## Results

### Overexpressing Myc during Larval Development Increases the Frequency of Somatic Mutations

**Figure 1:**
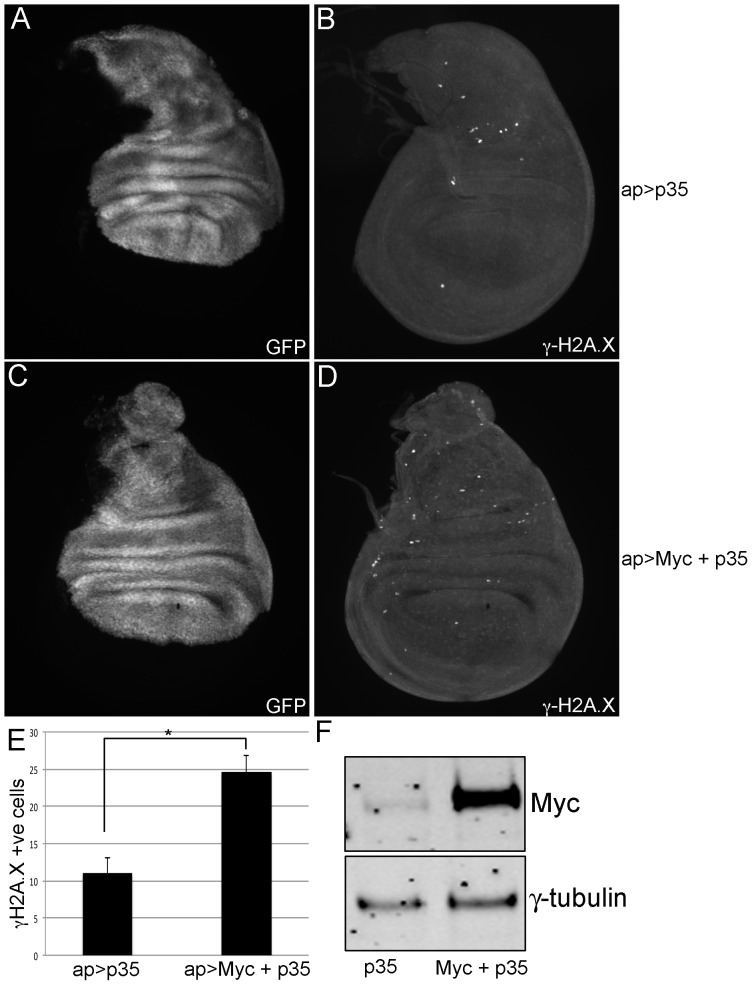
Myc overexpression increases the number of γ-H2A.X positive cells. (A, B) Wing imaginal disc of genotype ap-Gal4, UAS-GFP/+; UAS-p35/+. (C, D) Wing imaginal disc of genotype ap-Gal4, UAS-GFP/+; UAS-p35/UAS-Myc. GFP channel is shown in A and C, whereas γ-H2A.X staining is shown in B and D. (E) Quantitation of the number of γ-H2A.X positive cells from ap-Gal4, UAS-GFP/+; UAS-p35/+ wing discs (shown as ap>p35) and ap-Gal4, UAS-GFP/+; UAS-p35/UAS-Myc (shown as ap>Myc+p35). The number of γ-H2A.X positive cells within the ap>Gal4 expressing region (shown in GFP channel) from at least 10 imaginal discs was quantitated, and the error bars represent standard error. *indicates statistical significance of p<0.01 (student’s t-test). (F) Western blot analyses showing levels of Myc and the loading control γ-tubulin of wing imaginal discs of the genotypes ap-Gal4, UAS-GFP/+; UAS-p35/+ (labeled as p35) and ap-Gal4, UAS-GFP/+; UAS-p35/UAS-Myc (labeled as Myc+p35). Eight discs were loaded per lane. Myc is overexpressed 5.3-fold as quantitated using LiCOR software and normalized to the level of γ-tubulin (average of three Westerns).

In mammalian cells, overexpression of c-Myc increases the number of DNA double strand breaks (DSBs), as assayed by the level of γ-H2A.X, a phospho-histone marker of DSBs [9], [11]–[13], [15]. To determine if this is a conserved feature of Myc family proteins, we overexpressed *Drosophila* Myc in the dorsal compartment of the larval wing imaginal disc using apterous-Gal4 (ap-Gal4) and showed that this led to a ∼2-fold increase in the number of γ-H2A.X positive cells (Figure S1). Myc overexpression can cause apoptosis in wing disc and other mitotically dividing (but not endoreplicating) cells, which also induces phosphorylation of H2A.X [30], [41]. We therefore co-expressed Myc with the cell death inhibitor p35 and re-quantified the number of γ-H2A.X positive cells. Jointly overexpressing Myc and p35 increases the number of γ-H2A.X positive cells ∼2.5-fold compared to overexpression of p35 alone (Figure 1). Excess Myc therefore indirectly or directly increases the frequency of DSBs.

**Figure 2:**
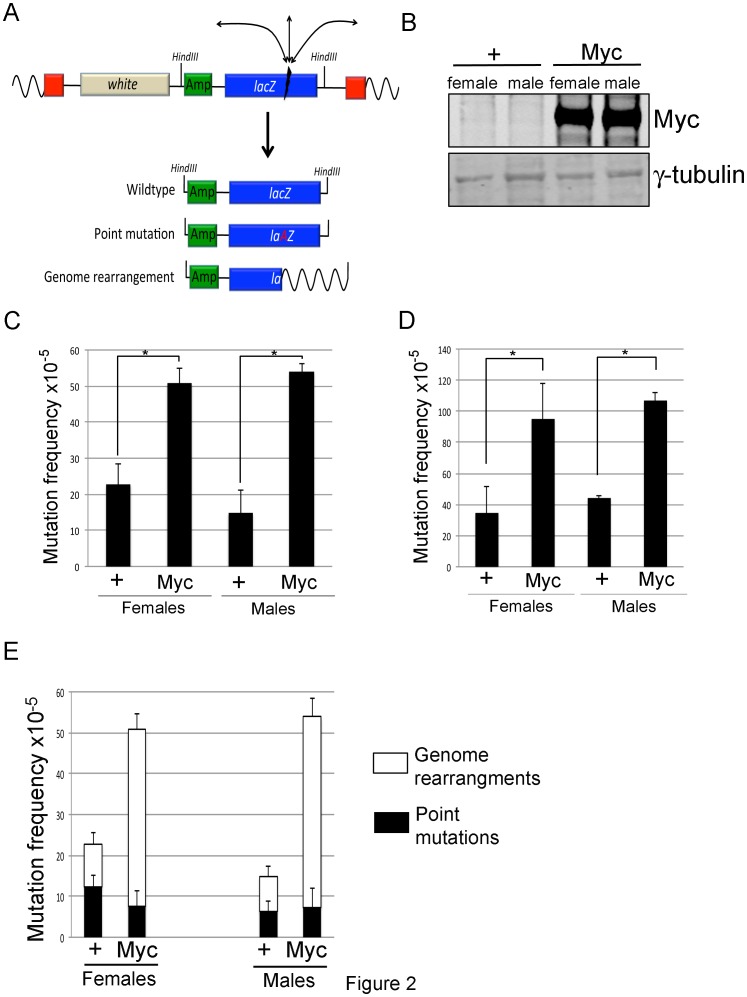
Myc overexpression increases mutation frequency. (A) Schematic representation of the *lacZ* reporter we used to quantitate mutation frequency, and potential mutation events that can occur in this transgene. Adapted from Garcia et al. [29] (B) Western analyses showing 9-fold overexpression of Myc compared to the loading control of γ-tubulin. Samples are wing imaginal discs of the genotypes hs-FLP; *lacZ* #2/+; act>CD2>Gal4, UAS-GFP/+ (shown as “+”) and hs-FLP; *lacZ* #2/+; act>CD2>Gal4, UAS-GFP/UAS-Myc larvae (shown as “Myc”). Larvae were heat shocked (37°C) for 45 min during the 3^rd^ larval instar and allowed to develop for 12 hours. Eight discs were loaded per lane. (C) *lacZ* mutation frequency of hs-FLP; *lacZ* #2/+; act>CD2>Gal4, UAS-GFP/+ (shown as “+”) and hs-FLP; *lacZ* #2/+; act>CD2>Gal4, UAS-GFP/UAS-Myc larvae (shown as “Myc”). Larvae are collected 3 days after a 45 minute heat shock at 37°C during the 1^st^ instar larval stage and separated by sex. Experiments shown are from at least four biological repeats (D) *lacZ* mutation frequency of hs-FLP; *lacZ* #9/+; act>CD2>Gal4, UAS-GFP/+ (shown as “+”) and hs-FLP; *lacZ* #9/+; act>CD2>Gal4, UAS-GFP/UAS-Myc (shown as “Myc”) larvae. (E) A selection (at least 48) plasmids were chosen to digest with AvaI to determine plasmid size per experiment for hs-FLP; *lacZ* #2/+; act>CD2>Gal4, UAS-GFP/+ (shown as “+”) and hs-FLP; *lacZ* #2/+; act>CD2>Gal4, UAS-GFP/UAS-Myc (shown as “Myc”) larvae. Male and female data are shown separately for these analyses and do not show any differences in mutation spectra. White filled area of bar represents frequency of size change mutation (genome rearrangement), where as black solid area shows frequency of mutant plasmids that do not show a size change (point mutations). *indicates statistical significance of p<0.001 (student’s t-test).

To establish whether *Drosophila* Myc’s ability to increase the number of DSBs leads to an increased mutation load, we overexpressed Myc during larval development and assessed mutation frequency using an *in vivo* lacZ mutation reporter transgene (Figure 2A) [27], [29]. We ubiquitously overexpressed Myc using the UAS/Gal4 system and the established “FLP on” strategy [42]. By using a strong (45 min) heat shock during the first larval instar more than 90% of larval cells overexpress Myc as assessed by anti-Myc staining and by examining the number of GFP positive cells, which is co-expressed with Myc (data not shown). Using this system, Myc is overexpressed an average of 9-fold as assessed by quantitative anti-Myc Western blot (Figure 2B). Heat shocked animals were then allowed to develop into 3^rd^ instar larvae, at which point they were separated by sex, and their *lacZ* mutation frequency determined using total genomic DNA as described previously [27], [29]. Mutation frequencies are expressed per locus, i.e., the number of mutant *lacZ* genes over the number of *lacZ* copies recovered from a DNA sample. As shown in Figure 2C, the spontaneous mutation frequency of both male and female larvae overexpressing Myc is approximately double that of control larvae that do not contain a UAS-Myc transgene. Myc overexpression during larval development therefore increases mutation load.

Our initial mutation analysis was carried out using a *lacZ* reporter inserted at cytological location 36F on the 2^nd^ chromosome (*lacZ* #2) [29]. To ensure that our increased mutation frequency was not specific to this genomic location, we quantitated the mutation frequency of control and Myc overexpressing larvae carrying an independent *lacZ* reporter inserted at 46D on the 2^nd^ chromosome (*lacZ* line #9) [29]. As shown in Figure 2D, although the baseline number of spontaneous mutations is higher at this genomic locus [29], both male and female larvae overexpressing Myc show a ∼2-fold increase in the number of mutations in the *lacZ* reporter. Myc therefore increases the mutation load at more than one genomic locus, so is likely to influence the frequency of mutations genome-wide.

### Myc Overexpression Increases the Severity of DSB-induced Mutations

We next assessed the type of mutation induced by Myc overexpression during larval development. This classification is based on the fact that point mutations and small alterations to the *lacZ* gene will not change the size of the rescued plasmid, while larger deletions and inversions (genome rearrangements) alter the size of the plasmid (Figure 2A). Restriction enzyme digestion-based size analyses of rescued *lacZ*-containing plasmids results in two broad classes of mutation: point mutations and genome rearrangements [27]–[29]. As seen in Figure 2E, Myc specifically increases the number of genome rearrangements that are caused by erroneous repair of DNA double strand breaks in both male and female larvae.

In addition to quantifying the number and type of mutation caused by Myc overexpression, we also determined the precise molecular basis of *lacZ* reporter mutations from control and Myc overexpressing larvae. To do this, we sequenced a subset of *lacZ*-containing plasmids from control and Myc overexpressing larvae containing *lacZ* insertion #2. Because Myc overexpression increases the frequency of DSB-induced mutations and not point mutations, we restricted our sequence analyses to plasmids that were classified as genome rearrangements above (Figure 2E).

Sequencing our *lacZ*-containing plasmids revealed breakpoints in the *lacZ* gene, which we then mapped to the *Drosophila* genome or sequences within the *P*-element by BLAST searches. Based on these data, we classified our DSB-mediated mutations as either indels (insertions, deletions and inversions) or translocations (sequences from other chromosomes). As a measure of mutation severity, we subdivided our indels into “small” or “large”: small indels involve DNA sequences less than 10 kb from the *lacZ* breakpoint site, and large indels involve sequences more than 10 kb away. While the cutoff of 10 kb to distinguish small indel from large is arbitrary, it serves a useful purpose in comparing mutations found in control and Myc overexpressing larvae. For these analyses, we pooled samples from male and female larvae since both showed an increase in the frequency of DSB-mediated mutations. As seen in Table 1, 61.9% of spontaneous DSB-mediated mutation events in control *lacZ* #2-containing larvae were small indels and 28.6% were large. In contrast, in Myc overexpressing larvae only 19.2% of indels were small and the vast majority (69.3%) were large. The frequency of translocations does not vary significantly between control and Myc overexpressing larvae. From our sequence analyses of *lacZ* breakpoints, we also observe regions of microhomology in 85% of breakpoints in both control and Myc overexpressing larvae (Table S1). We have defined these regions of microhomology as stretches of at least 5 identical base pairs immediately flanking (within 50 bp) the breakpoint. While Myc and controls have regions of microhomology, the key difference is that while control animals repair their DSBs using sequences close to the original breakpoint, Myc overexpressing larvae repair using sequences that are more distant. Taken with our γ-H2A.X data, Myc overexpression increases both the frequency and the severity of spontaneous DSB-induced mutations.

**Table 1:** Myc increases the severity of spontaneous DSB-mediated mutations.

Larval genotype	% small indel (<10 kb)	% large indel (>10 kb)	% translocation
**+ (N = 21)**	61.9	28.6	9.5
**Myc (N = 26)**	19.2	69.3	11.5

Control larvae are labeled as “+” and are the genotype hs-FLP/+ (or Y); *lacZ* #2/+; actin>CD2>Gal4, UAS-GFP/+ and Myc overexpressing larvae are labeled “Myc” and are the genotype hs-FLP/+ (or Y); *lacZ* #2/+; actin>CD2>Gal4, UAS-GFP/UAS-Myc. N indicates the number of breakpoint events counted. “Indels” are insertion and deletion events within the *lacZ* gene and translocations involve sequence within the *lacZ* reporter gene from another chromosome. Small indels are defined as less then 10 kb and large indels are 10 kb or greater.

### Overexpression of Myc in Adults Increases Mutation Load and Shortens Lifespan

Accumulation of mutations, particularly genome rearrangements that are caused by the misrepair of DSBs, correlates with aging across metazoans [20], [22], [43], [44], although a causal link between these observations has not been established. We therefore tested whether overexpressing Myc increases mutation frequency in adults, and whether it affects adult lifespan. Because ubiquitous Myc overexpression during embryonic and/or larval development causes pupal lethality (data not shown), we generated a fly strain that combined a ubiquitously expressed Gal4 driver (Actin-Gal4) and a temperature sensitive form of the Gal80 inhibitor protein (Tubulin-Gal80^TS^; referred to as Act^TS^ when combined). At 18°C Act^TS^ is off (due to Gal80^TS^ activity), at 29°C the Gal80^TS^ repressor is inactive thus Act^TS^ is active, and at the in-between temperature of 25°C intermediate levels of expression are observed [45].

**Figure 3:**
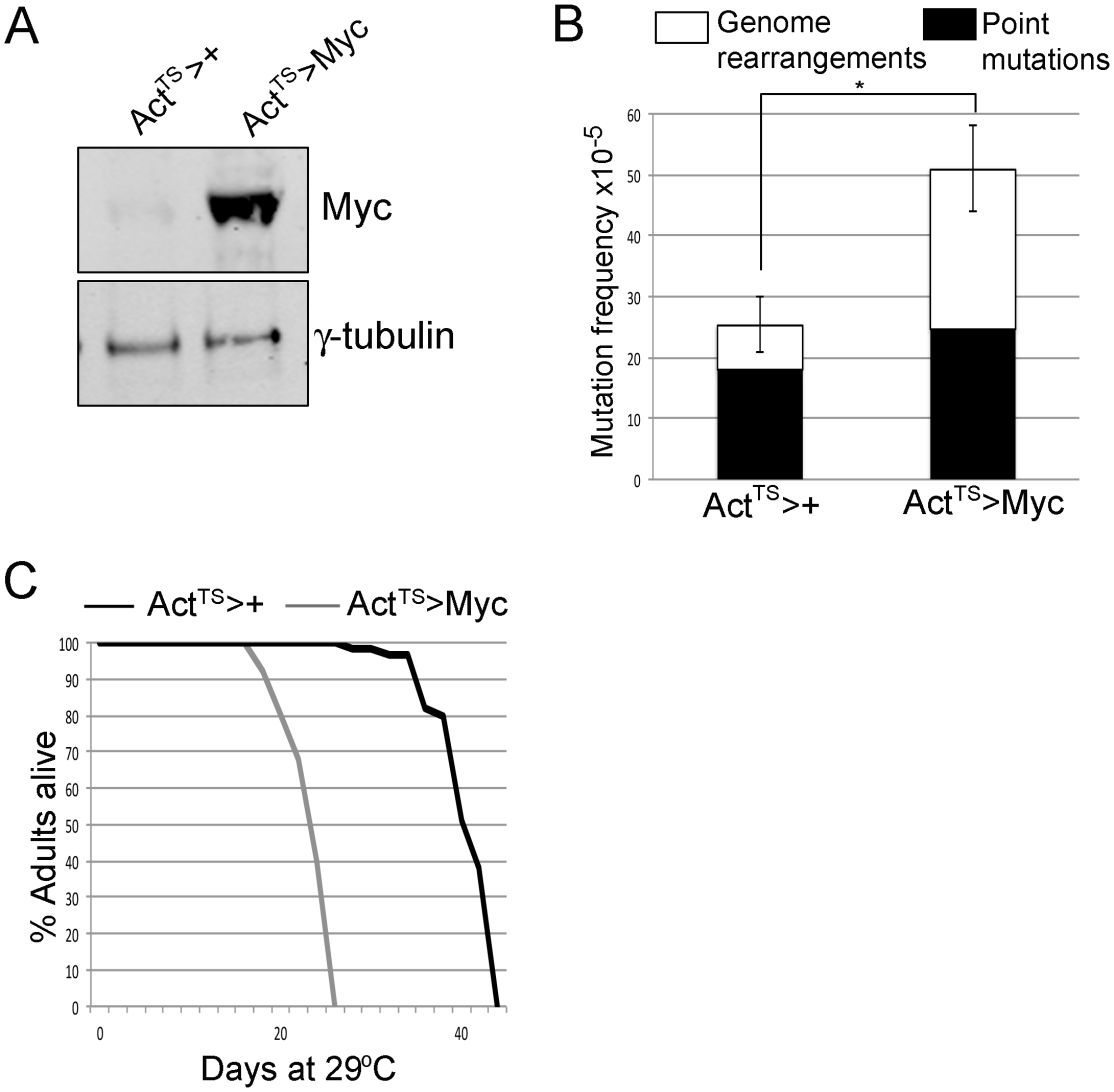
Overexpression of Myc increases mutation frequency and decreases lifespan at 29 °**C.** (A) Western blot from five adult heads at 29°C for three days from *lacZ* #2/Tub-Gal80^TS^; Actin-Gal4/+ (shown as Act^TS^>+) and *lacZ* #2/Tub-Gal80^TS^; Actin-Gal4/UAS-Myc (shown as Act^TS^>Myc). Myc is induced ∼9-fold in this system (LiCOR quantitation). (B) *lacZ* mutation frequency of *lacZ* #2/Tub-Gal80^TS^; Actin-Gal4/+ (shown as “Act^TS^>+”) and *lacZ* #2/Tub-Gal80^TS^; Actin-Gal4/UAS-Myc (shown as “Act^TS^>Myc”). Adults were placed at 29°C for 5 days before *lacZ* analyses to allow transgene expression. Black solid areas indicate frequency of point mutations and white areas indicate frequency of genome rearrangements. *indicates statistical significance of p<0.01 (student’s t-test). (C) Lifespan analyses of females of the genotype *lacZ* #2/Tub-Gal80^TS^; Actin-Gal4/+ (shown as “Act^TS^>+”) and of the genotype *lacZ* #2/Tub-Gal80^TS^; Actin-Gal4/UAS-Myc (shown as “Act^TS^>Myc”). These lifespans are statistically significantly different from each other p<0.001 (Log-rank test).

At 29°C, Myc levels are increased ∼9-fold using Act^TS^ compared to controls, and this increases the mutation frequency in adult females ∼2-fold after 5 days (Figure 3A, B). In addition, although we observe a higher proportion of point mutations in this genetic background than previously observed [29], Myc overexpression in adults increases the number of DSB-mediated genome rearrangements (Figure 3B). To assess whether overexpression of Myc in the adult affects lifespan, we used our Act^TS^ system to overexpress Myc in adult female flies and assessed their lifespan at 29°C where ∼9-fold induction of Myc is observed by Western analyses (Figure 3A). As shown in Figure 3C, Myc overexpression shortened median lifespan from 38 days in controls (Act^TS^ crossed to w^1118^) to 22 days.

**Figure 4:**
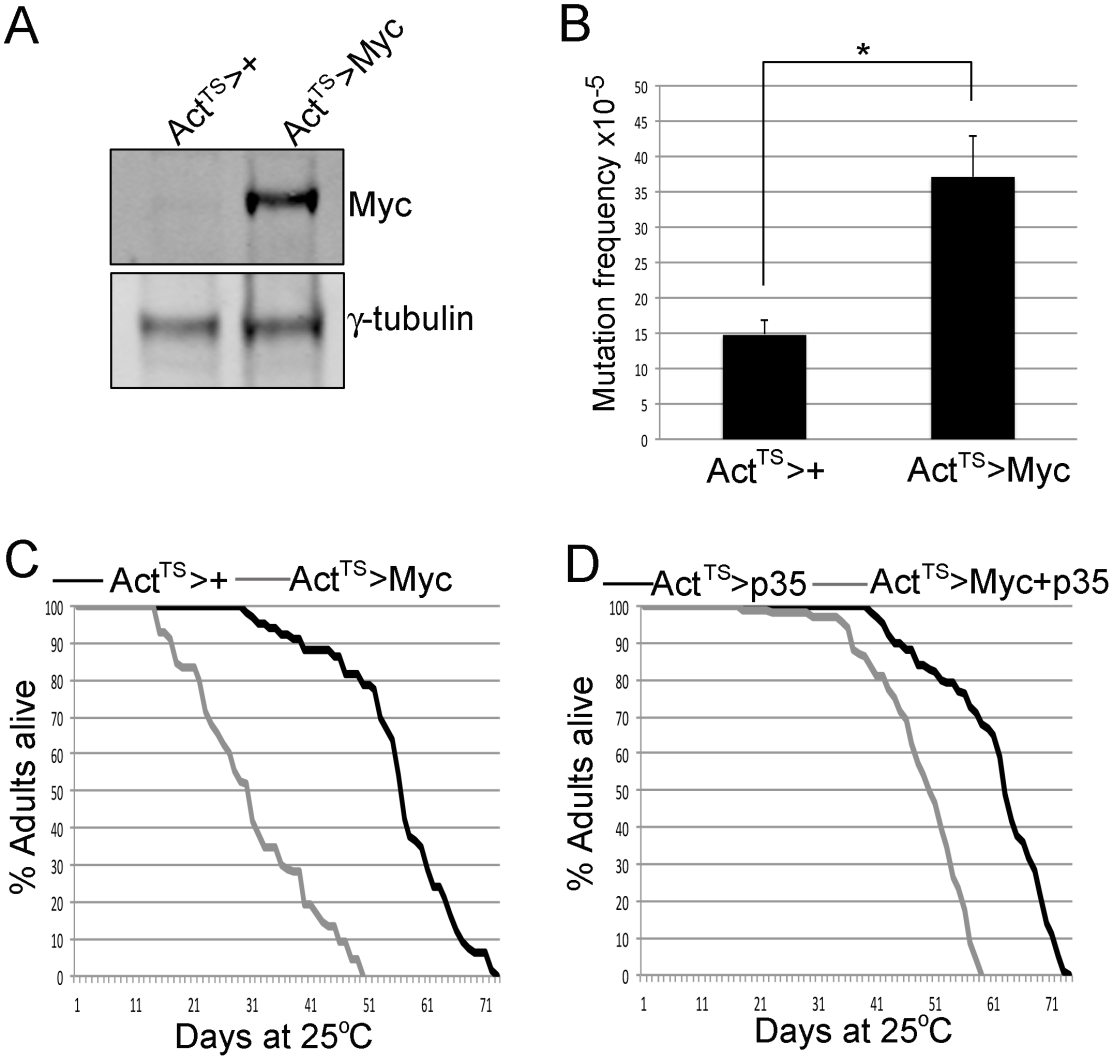
Myc overexpression in the adult increases mutation frequency and reduces lifespan at 25 °**C.** (A) Western analyses showing levels of Myc and the loading control γ-tubulin from four female adult heads of the genotypes *lacZ* #2/Tub-Gal80^TS^; Actin-Gal4/+ (shown as “Act^TS^>+”) and *lacZ* #2/Tub-Gal80^TS^; Actin-Gal4/UAS-Myc (shown as “Act^TS^>Myc”). Adults were placed at 25°C for 10 days before Western analyses. (B) *lacZ* mutation frequency of females of genotype *lacZ* #2/Tub-Gal80^TS^; Actin-Gal4/+ (shown as “Act^TS^>+”) and *lacZ* #2/Tub-Gal80^TS^; Actin-Gal4/UAS-Myc (shown as “Act^TS^>Myc”). Adults were placed at 25°C for 10 days before *lacZ* analyses. *indicates statistical significance of p<0.001 (student’s t-test). (C) Lifespan analyses at 25°C of females of the genotypes *lacZ* #2/Tub-Gal80^TS^; Actin-Gal4/+ (shown as “Act^TS^>+”) and *lacZ* #2/Tub-Gal80^TS^; Actin-Gal4/UAS-Myc (shown as “Act^TS^>Myc”). These lifespans are statistically significantly different from each other p<0.001 (Log-rank test). (D) Lifespan analyses at 25°C of females of the genotypes *lacZ* #2/Tub-Gal80^TS^; Actin-Gal4/UAS-p35 (shown as “Act^TS^>p35”) and *lacZ* #2/Tub-Gal80^TS^; Actin-Gal4/UAS-Myc, UAS-p35 (shown as “Act^TS^>Myc+p35”). These are statistically significantly different (p<0.001, Log-rank test).

Because 29°C is considered a stress temperature for flies, we also examined the lifespan of flies overexpressing Myc using Act^TS^ at 25°C, at which temperature we see a ∼5-fold upregulation of Myc protein levels (Figure 4A). Like Myc overexpression at 29°C, at 25°C we observe that Myc increases *lacZ* mutation frequency 2-fold after 10 days of expression (Figure 4B). As seen in Figure 4C, Myc overexpression shortens lifespan at 25°C from a median of 58 days to 31 days. It is not known whether Myc overexpression in adult flies causes apoptosis, but based on its ability to cause cell death in diploid (but not polyploid) larval cell types, it is possible that this occurs and could contribute to our observed Myc-induced shortened lifespan. To resolve this concern, we co-expressed Myc with the cell death inhibitor p35 and compared their lifespan to flies expressing p35 alone using Act^TS^. Overexpression of p35 has been previously shown to not significantly affect lifespan when expressed in the adult [46]. As shown in Figure 4D, flies co-expressing Myc and p35 have a longer median lifespan than flies expressing Myc alone, consistent with Myc-induced apoptosis in the adult influencing lifespan. However, flies co-expressing Myc and p35 still have a significantly shorter median lifespan than flies overexpressing p35 alone (50 days and 61 days, respectively). Thus, while Myc-induced cell death plays a part in the early death of Myc overexpressing flies, non-apoptotic functions also contribute. A good candidate for the early death of flies co-expressing Myc and p35 is an increased mutation frequency leading to cellular and tissue dysfunction. Importantly, at the non-permissive temperature of 18°C, we do not see any increase in Myc protein levels, nor do we observe any changes to lifespan (Figure S2).

### Myc haploinsufficiency Lowers Mutation Frequency and Extends Lifespan

**Figure 5:**
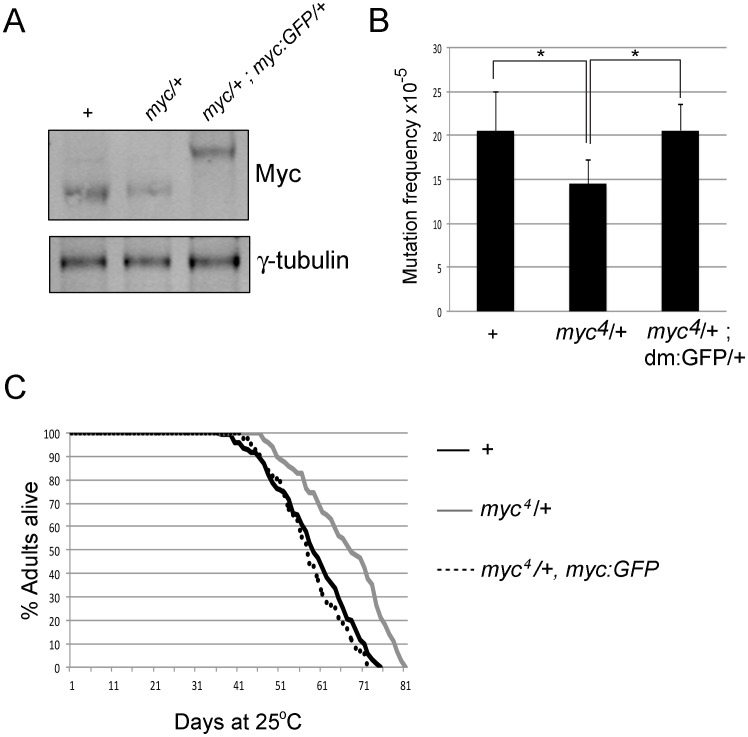
*myc* heterozygotes have a reduced mutation frequency and increased lifespan. (A) Western analyses showing levels of Myc and the loading control γ-tubulin from 20 female adult heads of the genotypes +/+; *lacZ* #2/+ (labeled as +), *myc^4^*/+; *lacZ* #2/+ (labeled as *myc*/+) and *myc^4^*/+; *lacZ* #2/+; *myc:GFP*/+ (labeled as *myc*/+, *myc:GFP*). Adults were 1–4 days old. (B) *lacZ* mutation frequency of +/+; *lacZ* #2/+ (labeled as +) and *myc^4^*/+; *lacZ* #2/+ (labeled as *myc^4^*/+) and *myc^4^*/+; *lacZ* #2/+; myc:GFP/+ (labeled *myc^4^*/+; myc:GFP/+). *indicates p<0.05 (student’s t-test). (C) Lifespan analyses of +/+; *lacZ* #2/+ (labeled as +), *myc^4^*/+; *lacZ* #2/+ (labeled as *myc^4^*/+) and *myc^4^*/+; *lacZ* #2/+; *myc:GFP*/+ (labeled as *myc^4^*/+, *myc:GFP*). The lifespan of *myc^4^*/+; *lacZ* #2/+ heterozygotes is significantly different to +/+; *lacZ* #2/+ and *myc^4^*/+; *lacZ* #2/+; *myc:GFP*/+ (which are indistinguishable) p = 0.002 (Log-rank test).

Overexpression of Myc family proteins increases genomic instability [6], [9], [12], [24]–[26], [47]. While understanding the effects of overexpressed Myc has clear relevance for understanding diseases caused by dysregulation of Myc such as cancer, we also wish to test a role for endogenous Myc in influencing mutation load. The *Drosophila* Myc gene (*myc,* also known as *diminutive* and *dm*) is located on the X chromosome and null alleles are hemizygous lethal [31]. We therefore examined flies with reduced Myc levels using females heterozygous for the null allele *myc^4^* and assessed their mutation load using our *lacZ* #2 reporter. *myc^4^* heterozygotes have a 2-fold decrease in Myc protein levels and have a significantly reduced *lacZ* mutation load (Figure 5A, B).Endogenous Myc therefore influences mutation frequency. Consistent with our data linking Myc overexpression to DSB-mediated mutations, categorizing the *lacZ* mutations found in *myc* heterozygotes revealed a decrease in the incidence of genome rearrangements (Figure S3). Thus the frequency of DSB-mediated mutations is increased by Myc overexpression and decreased by reducing Myc.

Because we observed a decreased mutation load in *myc^4^* heterozygous flies, we tested whether flies with reduced Myc levels also have an altered lifespan. We compared the lifespan of female flies heterozygous for *lacZ* #2 and those heterozygous for both *myc^4^* and *lacZ* #2. As shown in Figure 5C, flies heterozygous for *myc* and *lacZ* #2 live longer than *lacZ* #2 heterozygotes alone, living a median lifespan of 67 and 59 days, respectively. To confirm that this effect is specific to reduced Myc function, we used a fly strain harboring a transgene in which a GFP tagged version of genomic Myc is expressed under the control of its endogenous promoter. We crossed this transgene into the *myc^4^* null background and found that it is expressed at wildtype levels and rescues *myc^4^*-associated lethality, demonstrating that this transgene contains all the essential regulatory elements for *myc* expression (Figure 5A; data not shown). We then assessed the *lacZ* mutation load and lifespan of females flies heterozygous for *myc^4^*, *lacZ* #2 and the Myc:GFP rescue transgene. These flies have two copies of *myc* gene and are therefore expected to behave like wildtype flies. The decreased mutation load observed in *myc^4^* heterozygotes is restored to wildtype levels by the addition of one copy of the *myc:GFP* transgene (Figure 5B). In addition, flies heterozygous for *myc^4^*, *lacZ* #2 and *myc:GFP* have a median lifespan indistinguishable from *lacZ* #2 heterozygous controls (58 days; Figure 5C). Lowering Myc levels therefore reduces mutation load and extends lifespan.

## Discussion

Here we describe the first molecular characterization of mutations induced by overexpression of the *Drosophila* ortholog of the human Myc family of oncoproteins (c-Myc, N-Myc and L-Myc). Using an *in vivo lacZ* reporter, we demonstrate that Myc overexpression during larval development doubles *lacZ* mutation load at two different genomic locations. The increased mutation load observed in Myc overexpressing animals is due to an increase in the number of DSB-induced genomic rearrangements and they are more severe than those observed in control animals. We also correlate Myc-dependent changes in mutation load to changes in lifespan. Overexpression of Myc during adulthood increases mutation frequency and shortens lifespan and, conversely, decreasing Myc reduces mutation load and lengthens lifespan. These data implicate Myc as a possible new pro-aging factor.

Using the same *lacZ* reporter used in our study, changes in mutation load and lifespan have been recently observed in flies mutant for the bloom syndrome (BLM) DNA repair helicase [48]. Because dysregulation of Myc and BLM are both linked to cancer in humans [1], [49], we compared the molecular nature of mutations induced by these two genes in flies. While Myc overexpression and loss of BLM in flies both increase the number of DSB-induced genome rearrangements that are hallmarks of transformed cells, the precise nature of the mutations induced in these two contexts is different. Mutations induced by Myc overexpression were primarily large indels that involved sequences from outside the *lacZ* reporter. While Myc-induced mutations were more severe than those found in control larvae, their breakpoints did not show any distinguishing characteristics: They did not show any preference for particular sequences or genic region (intron, promoter etc), nor did they occur in the vicinity of any canonical Myc/Max binding sites (CACGTG). In contrast, the genome rearrangements found in *blm* mutant flies showed a strong preference for highly repetitive sequences that could not be uniquely mapped back to the *Drosophila* genome. This suggests that while loss of mammalian BLM and overexpression of Myc both result in genomic instability and cancer, the mechanisms by which they do so are distinct. Tumors caused by misregulation of these genes are likely to have different characteristics and respond very differently to drug therapies.

While Myc-induced mutation breakpoints do not appear to show any specific sequence preference like *blm* mutants, we do find regions of microhomology in lesions from both control and Myc overexpressing larvae. While the term microhomology does not require a specific length of sequence identity, we have defined it as a stretch of at least five identical base pairs (within 50 base pairs) on each side of the repaired DSB-induced breakpoint. These regions of microhomology suggest that DSBs in larvae may be repaired by microhomology-mediated end joining (MMEJ), an end joining mechanism that utilizes small regions of homology (5–25nt) [50]. What is surprising is that while 85% of control and Myc-induced breakpoints have regions of microhomology, the mutations in Myc overexpressing larvae are more severe than in controls. This suggests that Myc alters or interferes with the repair of DSBs. Significantly, our observation that excess Myc changes the position of repair to generate more severe lesions may be integral to the ability of Myc to transform cells.

Many questions still remain regarding the mechanisms by which Myc overexpression increases genomic instability. To-date, a conflicting literature from cultured mammalian cells and cell-free systems have complicated our understanding of c-Myc’s ability to induce DSBs. For example, in different culture conditions, c-Myc overexpression increases the number of DSBs via reactive oxygen species (ROS)-dependent and independent mechanisms [25], [26]. Independently of ROS, Myc has been suggested to induce genomic instability via replication stress based on its ability to accelerate S phase and induce DSBs in a cell free system [6], [51]. However, replication stress is also not the sole means by which Myc can cause DNA damage, as Myc can cause DSBs in G0/G1 arrested cells [26]. Myc may therefore use different mechanisms to cause DSBs in different cellular conditions. Alternatively, the non-physiological conditions for these experiments may have caused misleading results. A systematic genetic approach using our *lacZ* reporter in a physiologically relevant animal model such as *Drosophila* will allow us to dissect the mechanism(s) by which Myc overexpression directly or indirectly causes DSBs. Indeed, we would argue that Myc may affect both the frequency of DSBs and how they are repaired.

Based on the correlation between the accumulation of DSB-mediated mutations and accelerated aging [22], we examined the effect of modulating Myc levels on *Drosophila* lifespan. We observe that Myc overexpression increases the frequency of DSB-mediated mutations and shortens lifespan. We also show that this is not because Myc overexpression simply induces apoptosis in the adult, since flies co-expressing Myc and the cell death inhibitor p35 still show a shortened lifespan. Oncogene-induced senescence can occur because replication stress triggers the DNA damage response [52]. Our Myc-overexpressing flies may therefore undergo premature DNA damage-mediated senescence, leading to a shortened lifespan. It is also possible that the Myc-induced shortened lifespan is not specifically due to their increased mutation load. Because Myc function remains largely unexplored in the adult, is possible that one of its other functions (e.g. induction of cell growth) could decrease lifespan. If Myc’s regulation of cellular growth rate is key to its ability to modulate lifespan, it is tempting to suggest that this may be via the Insulin (IIS) signaling pathway that has been previously implicated in aging across a number of species [53]. Myc can regulate the IIS downstream target gene eIF4E in mammalian cells [54], [55], and has been linked to both Foxo and TOR in flies, although this appears to be highly tissue-specific [56], [57]. Myc may therefore be a new component to this highly conserved lifespan/aging pathway.

Complementing our Myc overexpression analyses, we also provide evidence for an endogenous role for Myc in modulating mutation load and lifespan. Females heterozygous for a null mutation in *myc* (*myc^4^*) [31] have a decreased mutation frequency and a longer lifespan than control animals. In flies, overexpression of Myc accelerates S-phase [41], [58], which is associated with replication stress and the generation of DSBs [6]. Reducing Myc levels by half may therefore slow replication, allowing more accurate repair of spontaneously induced mutations and slowing the accumulation of DNA damage.

Taken together, our analyses show that Myc increases the frequency and the severity of DSB-mediated mutations in *Drosophila*. Importantly, we are the first to show modulating Myc levels influences lifespan. These data therefore have significant implications for our understanding of how Myc-mediated changes to mutation load affect aging. Because genomic instability is also a hallmark of cancer, understanding the link between Myc levels and mutation load is also key to understanding the many functions of Myc that, when combined, lead to its potent oncogenic capability.

## Supporting Information

Figure S1 **Myc overexpression increases the number of γ-H2A.X positive cells.** (A, B) Wing imaginal disc of genotype ap-Gal4, UAS-GFP/+; +/+. (C, D) Wing imaginal disc of genotype ap-Gal4, UAS-GFP/+; +/UAS-Myc. GFP channel is shown in A and C, and γ-H2A.X staining is shown in B and D. (E) Quantitation of the number of γ-H2A.X positive cells from ap-Gal4, UAS-GFP/+; +/+ wing discs (shown as ap>+) and ap-Gal4, UAS-GFP/+; +/UAS-Myc (shown as ap>Myc). The number of γ-H2A.X positive cells within the ap-Gal4 expressing region (marked by GFP) were quantitated from at least 10 imaginal discs, and the error bars represent standard error. *indicates statistical significance of p<0.01 (student’s t-test). (F) Western blot analyses of Myc and the loading control γ-tubulin from 8 wing imaginal discs of the appropriate genotype showing levels of overexpression.
(TIF)

Figure S2 **Myc is not overexpressed and lifespan is unaltered at 18**°**C.** (A) Western blot from 20 female adult heads at 18°C for five days from *lacZ* #2/Tub-Gal80^TS^; Actin-Gal4/+ (shown as Act^TS^>+) and *lacZ* #2/Tub-Gal80^TS^; Actin-Gal4/UAS-Myc (shown as Act^TS^>Myc). Myc is not induced at this temperature (LiCOR quantitation). (B) Lifespan analyses at 18°C C of females of the genotype *lacZ* #2/Tub-Gal80^TS^; Actin-Gal4/+ (shown as “Act^TS^>+”) and of the genotype *lacZ* #2/Tub-Gal80^TS^; Actin-Gal4/UAS-Myc (shown as “Act^TS^>Myc”). These are not statistically significantly different (p>>0.05, Log-rank test).
(TIF)

Figure S3 ***myc^4^*** **heterozygotes have reduced levels of genome-rearrangement mutations.** (A) *lacZ* mutation frequency of females of the genotypes +/+; *lacZ* #2/+ (labeled as +) and *myc^4^*/+; *lacZ* #2/+ (labeled as *myc^4^*/+). Black solid areas indicate frequency of point mutations and white areas indicate genome rearrangements. *indicates statistical significance of p<0.05 (student’s t-test).
(TIF)

Table S1 **Molecular analyses of DSB-mediated breakpoints in control and Myc overexpressing larvae.** Genotype shown as “+” is hs-FLP/+ (or Y); *lacZ* #2; actin>CD2>Gal4, UAS-GFP/+ and genotype shown as “Myc” is hs-FLP/+ (or Y); *lacZ* #2; actin>CD2>Gal4, UAS-GFP/UAS-Myc. Sequence analyses was performed on a subset of plasmids previously defined as “genome rearrangements”. 21 events in control flies and 26 events in Myc overexpressing flies were found and characterized as either indels (insertion or deletion events) or as translocations (which involve sequence from another chromosome). Indels were defined as “small” (less then 10 kb) or “large” (10 kb or larger) based on our arbitrary cutoff. 50 base pairs on either side of each break point repair are shown. Microhomology is defined as 5 or more identical base pairs on either side of the breakpoint and is highlighted in bold. Locations and positions of break points are given as 5′ and 3′ unless they could not be determined from the sequence. Distance was calculated for all indels in base pairs from the breakpoint in *lacZ*. ^1.^ Location of the breakpoint or position of the event when inside the *lacZ* reporter. ^2.^ Position of the breakpoint(s) in the *lacZ* gene [27], [29]. ^3.^ Position of the breakpoint in the *Drosophila* genome as inferred from the 3′ genomic sequence captured in the *lacZ* reporter [27], [29]. ^4.^ Location of the breakpoint in the *Drosophila* genome determined by BLAST and FlyBase gene identity. ^5.^ Distance between the breakpoint in *lacZ* and the genomic position of adjoining piece of DNA in base pairs.
(XLSX)
