# Supplementary figures and images for: Myc-Dependent Genome Instability and Lifespan in Drosophila
Source: PLoS One. 2013 Sep 6;8(9):e74641. doi: 10.1371/journal.pone.0074641 (PMC3765364; doi:10.1371/journal.pone.0074641)

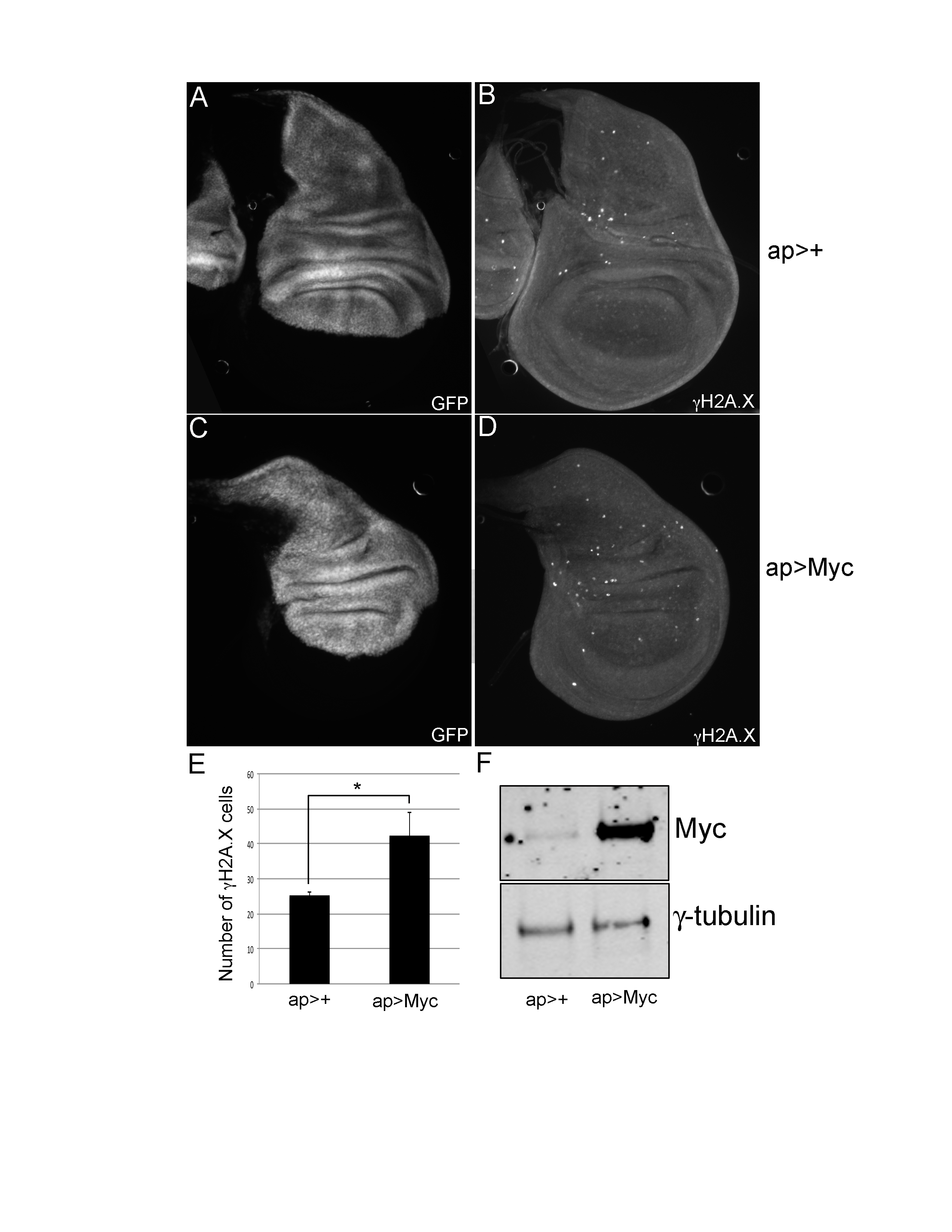

Supplement: Figure S1 — Myc overexpression increases the number of γ-H2A.X positive cells. (A, B) Wing imaginal disc of genotype ap-Gal4, UAS-GFP/+; +/+. (C, D) Wing imaginal disc of genotype ap-Gal4, UAS-GFP/+; +/UAS-Myc. GFP channel is shown in A and C, and γ-H2A.X staining is shown in B and D. (E) Quantitation of the number of γ-H2A.X positive cells from ap-Gal4, UAS-GFP/+; +/+ wing discs (shown as ap>+) and ap-Gal4, UAS-GFP/+; +/UAS-Myc (shown as ap>Myc). The number of γ-H2A.X positive cells within the ap-Gal4 expressing region (marked by GFP) were quantitated from at least 10 imaginal discs, and the error bars represent standard error. *indicates statistical significance of p<0.01 (student’s t-test). (F) Western blot analyses of Myc and the loading control γ-tubulin from 8 wing imaginal discs of the appropriate genotype showing levels of overexpression. (TIF) [file pone.0074641.s001.tif]

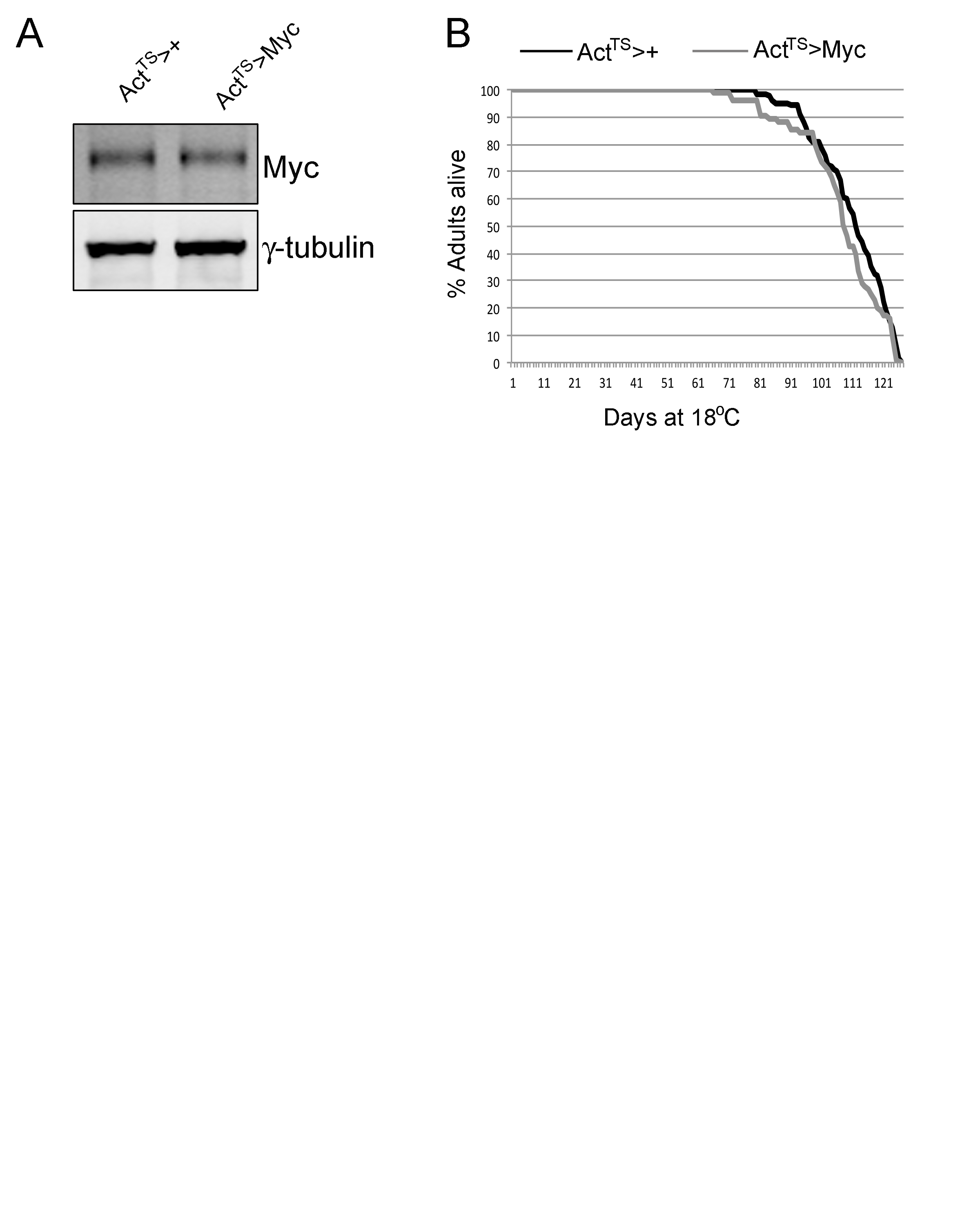

Supplement: Figure S2 — Myc is not overexpressed and lifespan is unaltered at 18°C. (A) Western blot from 20 female adult heads at 18°C for five days from lacZ #2/Tub-Gal80TS; Actin-Gal4/+ (shown as ActTS>+) and lacZ #2/Tub-Gal80TS; Actin-Gal4/UAS-Myc (shown as ActTS>Myc). Myc is not induced at this temperature (LiCOR quantitation). (B) Lifespan analyses at 18°C C of females of the genotype lacZ #2/Tub-Gal80TS; Actin-Gal4/+ (shown as “ActTS>+”) and of the genotype lacZ #2/Tub-Gal80TS; Actin-Gal4/UAS-Myc (shown as “ActTS>Myc”). These are not statistically significantly different (p>>0.05, Log-rank test). (TIF) [file pone.0074641.s002.tif]

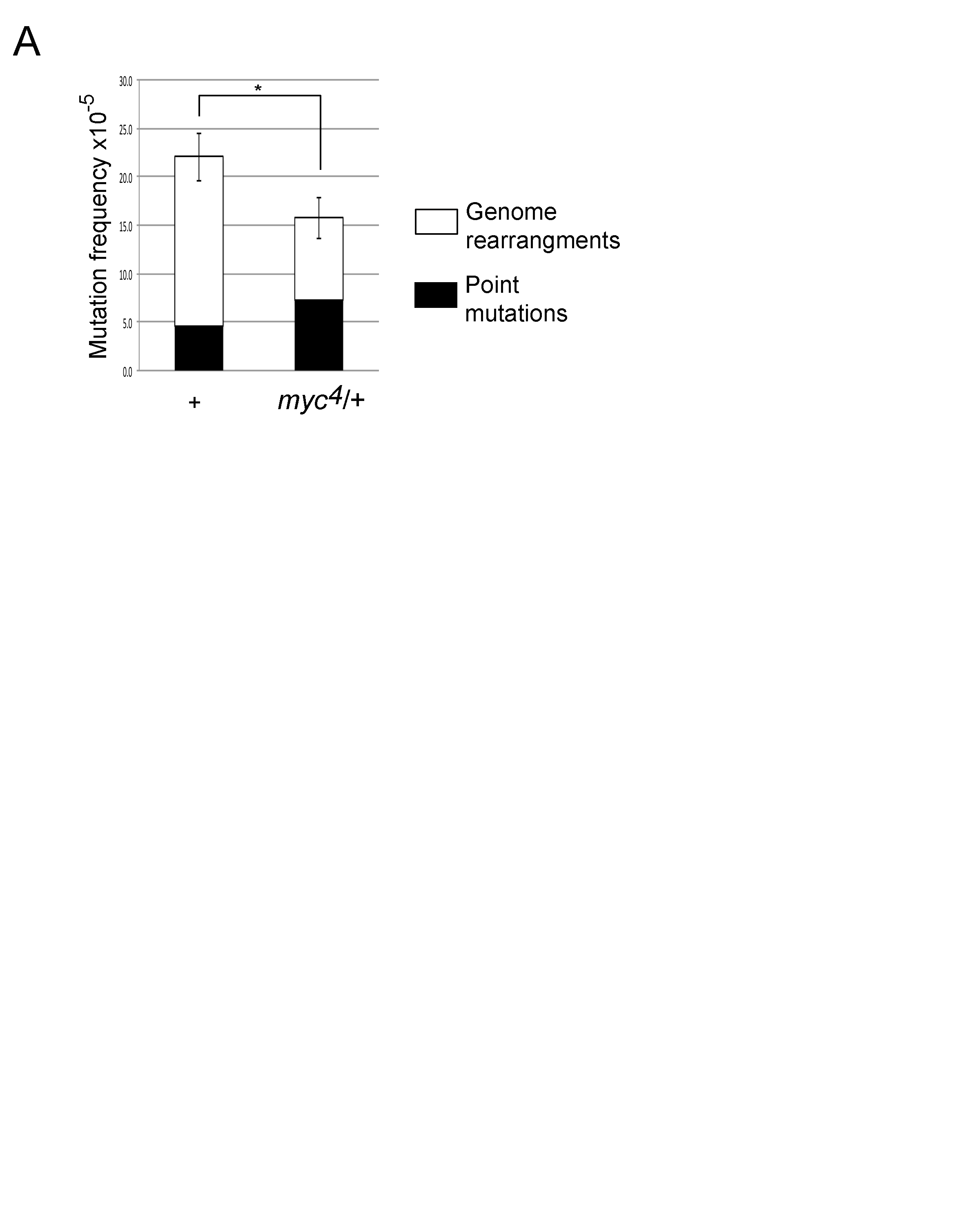

Supplement: Figure S3 — myc4 heterozygotes have reduced levels of genome-rearrangement mutations. (A) lacZ mutation frequency of females of the genotypes +/+; lacZ #2/+ (labeled as +) and myc4/+; lacZ #2/+ (labeled as myc4/+). Black solid areas indicate frequency of point mutations and white areas indicate genome rearrangements. *indicates statistical significance of p<0.05 (student’s t-test). (TIF) [file pone.0074641.s003.tif]
